# The Dangers of Unregulated Weight Loss Supplements: Tejocote-Induced Acute Liver Injury

**DOI:** 10.7759/cureus.57970

**Published:** 2024-04-10

**Authors:** Kyle Blackmer, Chris Elsayad

**Affiliations:** 1 Internal Medicine, Nassau University Medical Center, East Meadow, USA

**Keywords:** tejocotex, alipotec, drug-induced liver injury, weight loss, transaminitis, famotidine, tejocote root, tejocote

## Abstract

Tejocote, a product of the hawthorn tree and a common staple of Hispanic culinary practices and traditional medicine, has recently gained traction in the United States and Mexico as a means to achieve rapid effortless weight loss. This herbal supplement has largely evaded regulation by governing bodies in both countries despite documentation of several potentially serious adverse effects associated with its use. The present report describes the case of a middle-aged Hispanic female who ingested Tejocote with famotidine and presented with acute gastrointestinal symptoms and transaminitis, an unexpected symptom that warrants further investigation.

## Introduction

The continued worsening of the obesity pandemic has spurred increased demand for alternative, seemingly convenient weight loss methods, including over-the-counter herbal supplements not regulated by the Food and Drug Administration [1]. These compounds often possess wide-ranging side effect profiles with scant documented research. Tejocote, a product of the Mexican hawthorn tree *Crataegus mexicana*, is one such supplement marketed for weight loss. This report serves to review the potential adverse effects of this little-known herbal supplement and describe a novel drug-drug interaction experienced by our patient. In an era where patients are seeking alternative routes to weight loss than diet/exercise, it is crucial as healthcare providers to expand our understanding to include unregulated supplements such as tejocote so that we may proactively discuss pertinent risks and benefits and respond appropriately if symptoms arise.

## Case presentation

A 55-year-old obese Hispanic female with isolated asymptomatic dextrocardia and carpal tunnel syndrome presented to the emergency department with symptoms of nausea, abdominal pain, diarrhea, and decreased appetite. The patient stated she had been experiencing these worsening symptoms for the past month, partially relieved by famotidine. Vital signs were within normal ranges. No jaundice, tenderness to palpation, or hepatomegaly was noted on physical examination. Comprehensive metabolic panel was significant for potassium 3.2 mmol/L (3.6-5.2 mmol/L), aspartate aminotransferase (AST) 85 IU/L (8-48 IU/L), alanine aminotransferase (ALT) 107 IU/L (7-55 IU/L), alkaline phosphatase (ALP) 207 IU/L (40-129 IU/L). The electrocardiogram demonstrated normal sinus rhythm. Bilirubin levels were within normal ranges, Hepatitis serology was negative, and abdominal imaging results were unremarkable. She denied taking any prescription medications or using alcohol/illicit drugs recreationally. However, she reported starting an herbal supplement shortly prior to symptom onset. Further investigation revealed this supplement to be Tejocote - *Crataegus mexicana* - a Mexican root with purported weight loss benefits. She had stopped taking the supplement a few days before her presentation at the emergency department (ED) due to concerns it was related to her gastrointestinal (GI) symptoms. She was discharged in stable condition with instructions to avoid Tejocote and follow up with the outpatient clinic. Two weeks later, a follow-up visit to the outpatient primary care clinic revealed a complete resolution of transaminitis and all GI symptoms.

## Discussion

**Figure 1:**
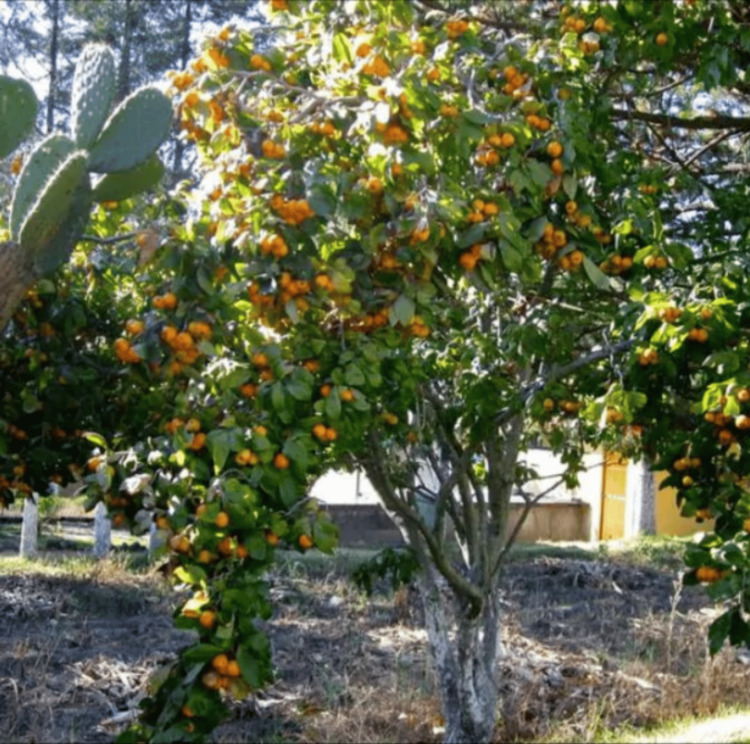
Crataegus mexicana tree laden with fruit Picture courtesy: https://www.quintadosouriques.com/store/seeds/trees/heart-plant-mexican-punch-tejocote-hawthorn-berry-small-mexican-apple/

**Figure 2:**
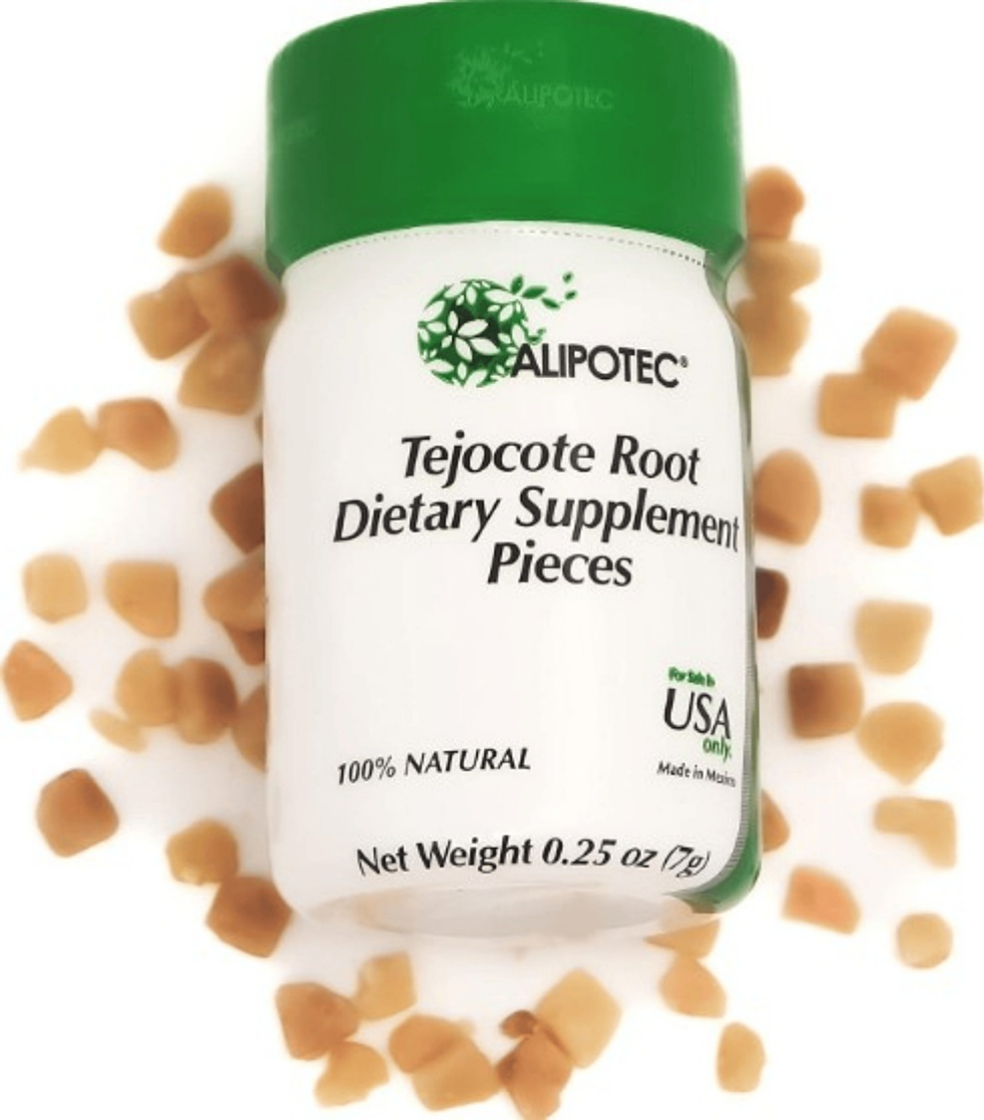
Tejecote root supplement Alipotec Picture courtesy: https://www.gosupps.com/tejocote-root-alipotec-classic-100-origin-complete-treatment-90-days.html Alipotec® (Alipotec, Mexico)

*Crataegus mexicana* is a species of Mexican hawthorn tree that bears fruits similar in size and taste to the common crabapple. The term “Tejocote” - from the Nahuatl word Texocotl (“stone fruit”) - directly refers to these small, round, yellow-orange fruits (Figure 1). They are traditionally used as pinata fillers, consumed directly, or used to make ponche (a brewed drink), marmalade, and other confections [2]. “Tejocote” also broadly refers to a pectin-rich preparation of the dried root of *C. mexicana* (marketed under the names Alipotec®/Tejocotex) that lowers appetite and promotes early satiety, leading to weight loss [3] (Figure 2).

**Figure 3:**
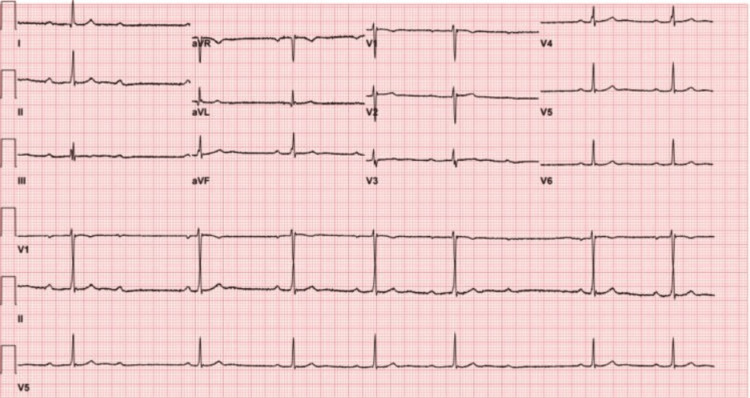
EKG demonstrating digoxin-like effects in a patient who consumed Tejocote Electrocardiogram (EKG) demonstrating digoxin-like effects (sinus bradycardia, 2nd-degree AV block) in a patient who consumed Tejocote. Used with permission from [5].

Gastrointestinal, cardiovascular, and hematologic effects of Tejocote have been previously documented, with GI effects being the most commonly reported [3]. These GI effects include varying degrees of abdominal pain, nausea, vomiting, diarrhea, and upper GI bleeding (some of which were observed in our patient). The GI effects of Tejocote likely arise from irritation of the gastric mucosal lining caused by the polyphenolic compounds (procyanidins, flavonols, flavones, and tannins) contained within *C. mexicana *[3]. Cardiovascular effects include positive inotropy, bradycardia, hyperkalemia, and second-degree atrioventricular (AV) block (Mobitz Type I). Some of the polyphenolic compounds responsible for the GI effects are structural analogues to cardiac glycosides, inhibiting the sodium-potassium pump and mimicking the cardiovascular effects of Digoxin (falsely elevated Digoxin levels have been documented in those consuming Tejocote) [4,5] (Figure 3). Hematologic effects include drug-induced immune thrombocytopenia that resolves upon cessation of Tejocote [6].

No documented literature attributes the consumption of *C. mexicana* to acute transaminitis or hepatotoxicity. In fact, studies demonstrate the flavonoids and phenolic acids contained within the plant possess antioxidant properties, providing hepatoprotective benefits against free radicals and reactive oxygen species [2,7,8]. Famotidine, taken alone, has been implicated as a rare cause of temporary drug-induced liver injury due to its activation into a toxic metabolite via the CYP450 system [9]; however, this requires chronic use at higher doses that did not occur with our patient. According to Basheer and Kerem, flavonoids, flavanols, anthocyanins, and tannins inhibit CYP3A4, decrease P-glycoprotein-mediated cellular efflux of drug metabolites, and inhibit intestinal glucuronidation of drug metabolites [10]. Thus, we postulate that ingestion of the polyphenolic compounds of Tejocote interfered with the drug metabolism of famotidine, resulting in the accumulation of hepatotoxic metabolites that induced acute transaminitis in our patient. It is also possible that, given the lack of literature on *C. mexicana*, the supplement may be directly hepatotoxic in rare cases or in individuals with certain CYP450 polymorphisms [11].

## Conclusions

Our patient presented with acute GI symptoms and elevated liver enzymes upon consuming Tejocote with famotidine; these symptoms rapidly resolved upon cessation of Tejocote. For healthcare providers, this case emphasizes the importance of obtaining a full patient medication history, including over-the-counter supplement use. Particular attention should be given to any acute rise in AST/ALT/ALP, digoxin-like side effects in patients not already taking digoxin (bradycardia, hyperkalemia, AV block, xanthopsia), thrombocytopenia, or acute nonspecific GI symptoms in a patient taking Tejocote or any other over-the-counter herbal remedy. It also highlights the importance of physician education on the potential adverse drug reactions of Tejocote and other unregulated compounds, so that we may pass this knowledge on to our patients and properly advise caution when warranted. Further research on the composition and metabolism of these clinically overlooked yet commonly consumed supplements is necessary to elucidate their full side effect profiles and drug-drug interactions.
